# Royal Medical and Chirurgical Society

**Published:** 1853-03

**Authors:** 


*Royal Medical and Chirurgical Society. On the Pathology of Rheumatic and Non-Rheumatic Pericarditis.* By Dr. Ormerod.

The author commenced by a reference to the researches of the late Dr. Taylor, who had satisfactorily shown that acute rheumatism was not exclusively the cause of pericarditis, and who had also called attention to the importance of granular disease of the kidney in reference to this morbid condition. The author desired to limit the use of the word pericarditis to present inflammation of the pericardium; and this analysis referred exclusively to classes of this nature. The means of investigation comprehended complete records of 1410 cases observed under nearly similar circumstances; that is, in the wards of different hospitals. Of these, 1249=88.59 per cent. were not cases of rheumatism; 161=11.41 per cent. were admitted on account of rheumatism, or suffered from it while under observation. Of the whole number,

85=6 per cent. had recent pericarditis, observed during life, or discovered after death, and were thus distributed:

| | | | |
|---|---|---|---|
| 24=1.92 per cent. | occurred among | 1249 | non-rheumatic cases. |
| 61=37.88 per cent. | " " | 161 | rheumatic cases. |
| 85=6 per cent. | | 1410 | |

The mean age of 61 subjects of rheumatic pericarditis was about 21; the mean age of 24 subjects of non-rheumatic pericarditis was 42; the extremes being 7 and 63 years. As to the different causes of the pericarditis:

| | |
|---|---|
| Rheumatic, ........ | 61 cases coincided with acute rheumatism. |
| Non-rheumatic of local origin, | 7 ensued on inflammation of lungs or pleura. |
| | 2 ensued on malignant disease of the pericardium. |
| | 1 ensued on old cardiac disease. |
| Non-rheumatic of constitutional origin, | 6 coincided with granular disease of kidney. |
| | 4 coincided with hæmorrhage or exhaustion. |
| | 2 coincided with scarlatina or erysipelas respectively. |
| | 2 were inexplicable. |
| | 85 |

The date of the accession of pericarditis was determined in 33 of the rheumatic cases. The mean of these observations gave the 105th day of the rheumatic attack as that on which the pericardial complication most commonly supervened. The question, whether a first or second attack of rheumatism was more likely to be accompanied by pericarditis, was beyond the reach of hospital statistics. This source of information was silent, also, on the question, whether pericarditis be more likely to occur in severe or in the slighter cases of rheumatic fever. It might, however, be safely inferred, that the severity of the articular and pericardial affections bore no very close relationship to each other. It was certain that the most severe, even fatal pericarditis, might occur where there was but faint evidence of articular affection, and this latter condition might exist in the most aggravated and intense form without involving the addition of pericarditis to the other sources of distress. The author then entered upon the consideration of the subject of non-rheumatic pericarditis of local origin; and a question of importance here presented itself—what was the influence of pre existent cardiac or pulmonary affections in inducing inflammation of the pericardium? The question was of equal importance in relation to acute rheumatism. The relation of pulmonary inflammation to pericarditis was thus illustrated: In the 1410 cases, the basis of this inquiry, some form of pulmonary inflammation—that is, pneumonia, pleuritis, or pleuro-pneumonia—was ascertained to exist, either by auscultation or dissection, in 265 cases. Of these:

| | | | | |
|---|---|---|---|---|
| 117 | had pneumonia, | of which | 19 | had recent pericarditis. |
| 86 | had pleurisy, | " | 6 | " |
| 62 | had pleuro-pneumonia, | " | 8 | " |
| 265 | | | 33 | = 12.4 per cent. |

In the rheumatic class, pericardial inflammation commonly preceded, yet sometimes, though rarely, followed pulmonary inflammation. The non-rheumatic class told quite a different story; here pulmonary inflammation had apparently a distinct influence in inducing pericarditis, and this influence was most evident in cases of pleurisy; and clinical observation bore out the conclusion, that the pericarditis was subsequent to, and probably contingent on, the pulmonary inflammation. The author then referred to the comparative fatality of non-rheumatic compared with rheumatic pericarditis, and also to the desirableness of instituting an exact comparison between Bright's disease of the kidney and acute rheumatism, in respect to their tendencies to induce inflammation of the pericardium. In conclusion, the author desired to ascertain how far the results obtained by his present analysis agreed with those of the published cases of Dr. Taylor, who had made the subject of non-rheumatic pericarditis so peculiarly his own. The deductions seemed identical, and one rose from the perusal of those elaborate clinical reports with a conviction that non-rheumatic pericarditis was more within the province of the anatomist than of the physician. It was a disease with few or no symptoms, its physical signs recognized more often by a chance discovery than on the suggestions of the disease, and its morbid changes small in amount and apparently inactive; and where opportunity had occurred of watching the disease some time previous to death, it had been apparently without effect on the general symptoms, its presence or absence being determined by the ear alone; and still, in these, its connection with the fatal termination had appeared to be that of a coincidence rather than of a cause.—*Dublin Medical Press.*

